# CCR7^lo^PD-1^hi^ CXCR5^+^ CD4^+^ T cells are positively correlated with levels of IL-21 in active and transitional cystic echinococcosis patients

**DOI:** 10.1186/s12879-015-1156-9

**Published:** 2015-10-26

**Authors:** Fengbo Zhang, Nannan Pang, Yuejie Zhu, Dexian Zhou, Hui Zhao, Jinwei Hu, Xiumin Ma, Jun Li, Hao Wen, Buka Samten, Haining Fan, Jianbing Ding

**Affiliations:** Department of Clinical Laboratory, First Affiliated Hospital of Xinjiang Medical University, 393 Xinyi Road, 830011, Urumqi, Xinjiang China; Department of Hepatopancreatobiliary Surgery, Affiliated Hospital of Qinghai University, 251 Xining Road, 810000, Xining, Qinghai China; Department of Pulmonary Immunology, University of Texas Health Science Center at Tyler, Texas, 75708, USA; Department of Immunology, School of Preclinical Medicine of Xinjiang Medical University, 393 Xinyi Road, 830011, Urumqi, Xinjiang China

**Keywords:** Cystic hydatid disease, Tfh cells, IgG antibodies, Cytokines, Bcl-6

## Abstract

**Background:**

In our study, we investigated whether circulating T follicular helper (Tfh) and the related cytokines are involved in human cystic echinococcosis (CE).

**Methods:**

A total of 64 patients with CE and 30 healthy controls were enrolled in this study. Percentages of CCR7^lo^PD-1^hi^ cells within CXCR5^+^ CD4^+^ T cells (circulating Tfh cells) were detected by flow cytometry. Levels of IL-21 and IL-4 in peripheral blood were detected by cytometric bead array. The mRNA expression of IL-21, IL-4, Bcl-6, and Blimp-1 in peripheral blood mononuclear cells (PBMCs) were measured by real-time PCR. Levels of IgG1, IgG2, IgG3, and IgG4 in the patients’ sera were measured using enzyme-linked immunosorbent assay.

**Results:**

Percentages of circulating Tfh cells were significantly increased in the CE1, CE2, and CE3 groups (*p* < 0.05). The concentrations of IL-21 and IL-4 in the serum were significantly increased in CE1, CE2, and CE3 groups (*p* < 0.05). IL-21 was positively correlated with circulating Tfh cells in CE3 group (*r* = 0.779, *p* < 0.05). The mRNA levels of IL-21, IL-4, and Bcl-6 were increased in CE1, CE2, and CE3 groups. Levels of IgG1 and IgG4 in patients’ sera were increased in CE1, CE2, and CE3 groups. Levels of IgG2 and IgG3 were increased in CE4-5 group. Additionally, after stimulation with hydatid fluid in vitro, the levels of circulating Tfh cells, IL-21 and IL-4 in PBMCs isolated from CE patients were significantly increased (*p* < 0.05).

**Conclusions:**

The levels of circulating Tfh and related cytokines were significantly increased in CE patients, suggesting that they are involved in human CE.

**Electronic supplementary material:**

The online version of this article (doi:10.1186/s12879-015-1156-9) contains supplementary material, which is available to authorized users.

## Background

Cystic echinococcosis (CE), also known as hydatidosis, is a zoonotic parasitic disease with a high incidence in Xinjiang, a pastoral area in China [1]. CE is caused by parasitic larvae of *Echinococcus granulosus* (*Eg*) in the intermediate hosts such as human and sheep [1–3]. Larvae of *Eg* have a bladder-like morphology and parasitize the parenchymas of internal organs, especially the liver and lungs. The larval stage of *Eg* forms a cyst that is filled with hydatid fluid (HF), and is surrounded by three membrane layers. Antigen B of *Eg* (AgB) is one of the major immunodominant antigens of HF [4]. Cysts can undergo structure changes during the progression of the disease. Based on the ultrasound image and morphological changes in the structure of hepatic cystic, CE is classified into CE1-2 (activity), CE3 (transition), and CE4-5 (inactivity) types [5, 6]. CE1-CE5 types are characterized by the appearance of cyst contents and wall. In CE4 and CE5, the viability of parasite tissue is very low, therefore, the CE4 and CE5 cyst are considered inactive. In CE1 and CE2, it is likely that cysts contain viable Protoscolices, thus, the CE1 and CE2 cysts are considered as active. The CE3 cysts show the collapse or detachment of the parent cyst wall [6].

The host immune responses to hydatid, especially antibody class switching, varies in different CE types. It was found that the positive rates of IgG4 in patient sera were increased in CE1, CE2, and CE3 types, but the positive rates of IgG1 and IgG4 were decreased in CE4–5 types [7]. Specific IgG1 and IgG4 against antigens of cyst fluid are dominant in CE with positive antibodies in sera [8]. IgG1, IgG4, IgE, and IgM are dominant in serum of patients with chronic infection, but with a relatively low level in the inactive stage of *Eg*-infection [7, 9]. The IgG1 and IgG4 are significantly decreased, but the IgG2 and IgG3 are significantly increased upon removal of hydatid cyst. Mechanism of the antibody class switching during hydatid disease is still unclear. Activities of Th1 and Th2 cells are involved in the chronic infectious parasitic diseases [10, 11]. Imbalance of Th1/Th2 exists in *Eg*-infection [12]. During the EG infection in humans, Th1 immune responses are dominant in the early stage of hydatid infection, and IL-2 and IFN-γ secreted by Th1 cells are directly related with the slow growth of hydatid. The Th2 immune responses become dominant in the advanced stages, associated with rapid growth of hydatid [13]. Activity of TGF-β/Smad signaling pathway results in up-regulation of IL-10 and TGF-β, and imbalance of Treg/Th17 [14]. Th9 cells are also involved in the immune responses against *Eg*-infection [15]. Relationship between the *Eg*-infection and the newly identified CD4^+^ T cells subgroup called follicular helper CD4^+^ T (Tfh) cells is not reported.

Previous studies suggested that the maturation and proliferation of B cell need the help of Tfh cells, which localize to the germinal center of B-cell follicle in secondary lymphoid organ [16, 17]. Tfh cells, the subset of CD4^+^ T cell expressing surface molecules of PD-1, CXCR5 and ICOS, play a central role in antibody generation and class switching. Tfh cells secrete the IL-4 and IL-21, cytokines that promote growth and differentiation of B cells. Bcl-6 that is strongly up-regulated in Tfh cells is identified as the master regulator of Tfh cells. The primary function of Bcl-6 in Tfh cells is to suppress the genes that are required to drive the differentiation of other Th cell lineages. Blimp-1, which antagonizes Bcl-6, can inhibit Tfh cell formation. Thus, Tfh cell generation is controlled by the balance of these two transcription factor. Without Tfh cells, germinal center is hardly formed and the humoral immune response by B cells is significantly decreased, leading to studies on mechanism of Tfh cells in humoral immune, such as virus infection, autoimmune disease and parasite infection [13, 18–24].

Circulating Tfh cell is considered as the counterpart of Tfh cells in the peripheral lymphoid organs [25–28]. Especially, He et al found that within circulating CXCR5^+^ CD4^+^ T cells, the CCR7^lo^PD-1^hi^ subset had a partial Tfh effector phenotype [25]. Thus, CCR7^lo^PD-1^hi^ cells within CXCR5^+^ CD4^+^ T cells in peripheral blood may serve as circulating Tfh cells. The role of the circulating Tfh cells in autoimmunity has been reported [20]. However, the role of circulating Tfh cell in the CE patients is not clear. Therefore, in the present study, we investigated the changes in percentages of circulating Tfh cells in peripheral blood of CE patients and the related cytokines and transcription factors. Correlation between circulating Tfh cells and antibody types of *Eg*-infection was also investigated.

## Methods

### Healthy individuals and patients

A total of 64 patients and 30 healthy individuals as controls were enrolled in this study and the detailed demographic information were included in the Table 1. No individuals in this study received anti-inflammatory drugs and patients with other diseases such as acute and chronic viral infections, autoimmune diseases, tumors, rheumatic diseases, sepsis, severe upper respiratory infections, lung or biliary tract infection, or fever were excluded. This study was approved by the Human Ethics Committee of the First Affiliated Hospital of Xinjiang Medical University (approval number: 20120220-126). All patients provided written informed consents.

**Table 1:** Information of the patients enrolled in this study

Case	Stages	Age (range)	Gender	Position	Size (cm)	Populations
1	CE1	21–25	Male	Left liver lobe	4.5	Han
2	CE1	21–25	Male	Right liver lobe	4.1	Han
3	CE1	21–25	Male	Left liver lobe	4.3	Han
4	CE1	31–35	Female	Right liver lobe	5.6	Uighur
5	CE1	36–40	Female	Right liver lobe	4.9	Kazak
6	CE1	26–30	Male	Right liver lobe	5.6	Uighur
7	CE1	26–30	Male	Right liver lobe	5.8	Uighur
8	CE1	21–25	Female	Left liver lobe	4.6	Han
9	CE1	21–25	Male	Left liver lobe	3.9	Kazak
10	CE1	36–40	Male	Left liver lobe	6.8	Uighur
11	CE1	26–30	Female	Right liver lobe	4.9	Uighur
12	CE1	31–35	Male	Left liver lobe	5.9	Uighur
13	CE1	31–35	Female	Right liver lobe	6.9	Han
14	CE1	36–40	Male	Right liver lobe	7.9	Uighur
15	CE1	41–45	Male	Right liver lobe	6.8	Uighur
16	CE1	36–40	Male	Right liver lobe	5.9	Kazak
17	CE2	36–40	Female	Right liver lobe	7.9	Kazak
18	CE2	36–40	Male	Left liver lobe	4.9	Han
19	CE2	46–50	Male	Left liver lobe	4.8	Kazak
20	CE2	31–35	Male	Right liver lobe	6.9	Kazak
21	CE2	36–40	Male	Left liver lobe	4.8	Uighur
22	CE2	31–35	Male	Right liver lobe	7.9	Han
23	CE2	21–25	Female	Right liver lobe	8.7	Han
24	CE2	31–35	Male	Left liver lobe	8.9	Han
25	CE2	26–30	Male	Right liver lobe, left liver lobe	8.6	Kazak
26	CE2	26–30	Male	Left liver lobe	8.9	Han
27	CE2	31–35	Male	Right liver lobe	8.7	Kazak
28	CE2	26–30	Female	Right liver lobe	9.4	Han
29	CE2	36–40	Female	Left liver lobe	10.6	Han
30	CE2	31–35	Male	Right liver lobe	11.9	Han
31	CE2	26–30	Female	Left liver lobe	15.6	Khalkhas
32	CE2	26–30	Male	Right liver lobe	16.3	Kazak
33	CE2	31–35	Male	Left liver lobe	18.9	Khalkhas
34	CE2	36–40	Female	Right liver lobe	19.6	Kazak
35	CE2	26–30	Male	Right liver lobe	20.9	Han
36	CE2	26–30	Female	Right liver lobe	21.6	Khalkhas
37	CE2	21–25	Female	Right liver lobe	12.3	Kazak
38	CE2	26–30	Male	Left liver lobe	14.4	Uighur
39	CE2	31–35	Male	Left liver lobe	7.6	Uighur
40	CE2	31–35	Female	Right liver lobe	5.6	Uighur
41	CE3	45–50	Female	Left liver lobe	7.8	Khalkhas
42	CE3	41–45	Female	Right liver lobe	19.3	Khalkhas
43	CE3	36–40	Male	Left liver lobe	12.3	Uighur
44	CE3	36–40	Female	Right liver lobe	14.2	Uighur
45	CE3	31–35	Male	Right liver lobe, left liver lobe	8.4	Han
46	CE3	26–30	Male	Left liver lobe	9.2	Tibetan
47	CE3	36–40	Male	Left liver lobe	8.6	Han
48	CE3	31–35	Female	Left liver lobe	7.6	Kazak
49	CE3	26–30	Male	Right liver lobe	6.4	Han
50	CE3	26–30	Male	Right liver lobe	12.3	Uighur
51	CE3	36–40	Male	Right liver lobe	11.4	Uighur
52	CE3	31–35	Male	Right liver lobe	8.3	Han
53	CE3	31–35	Male	Right liver lobe	6.8	Uighur
54	CE3	31–35	Male	Right liver lobe	11.6	Kazak
55	CE3	31–35	Female	Right liver lobe	6.8	Han
56	CE3	36–40	Female	Right liver lobe	7.9	Han
57	CE3	36–40	male	Right liver lobe	8.6	Han
58	CE4-5	36–40	Male	Right liver lobe	6.9	Han
59	CE4-5	41–45	Male	Right liver lobe	8.7	Han
60	CE4-5	41–45	Male	Right liver lobe	10.6	Mongolian
61	CE4-5	41–45	Male	Right liver lobe	5.6	Mongolian
62	CE4-5	36–40	Male	Right liver lobe	4.6	Uighur
63	CE4-5	36–40	Female	Right liver lobe	4.8	Mongolian
64	CE4-5	41–45	Male	Right liver lobe	9.4	Mongolian

### Real-time reverse transcription polymerase chain reaction (Real-time PCR)

Peripheral blood was collected from patients and healthy individuals after fasting for 8 h. Then peripheral blood mononuclear cells (PBMCs) were isolated and kept in liquid nitrogen for further analysis. Total RNA was extracted from PBMCs using Trizol LS reagent (Invitrogen, Life Technologies, USA) and reverse transcribed into cDNA by M-MLV Reverse Transcriptase (Invitrogen, Carlsbad, CA, USA). Primers were shown in Table 2. Real-time PCR was performed with the SYBR GREEN PCR Premix (TaKaRa Biotechnology CO., Ltd., Dalian, P.R. China) following the manufacturer's protocols. Target genes and β-actin were amplified. The target genes were quantified by Ct value using 2^-ΔΔCt^. Experiments were repeated for at least 3 times.

**Table 2:** Primers used in this study

Primers	Sequences (5' to 3')
IL-4_F	TTTGCTGCCTCCAAGAACAC
IL-4_R	TTCCTGTCGAGCCGTTTCAG
IL-21_F	ACACAGACTAACATGCCCTTCA
IL-21_R	ACCGTGAGTAACTAAGAAGCAAATC
BCL-6_F	GGAAACCCAGTCAGAGTATTCG
BCL-6_R	CACATTTGTAGGGCTTTTCTCC
β-actin_F	TAGGCGGACTGTTACTGAGC
β-actin_R	TGCTCCAACCAACTGCTGTC
Blimp-1_F	TCCAGCACTGTGAGGTTTCA
Blimp-1_R	TCAAACTCAGCCTCTGTCCA

### Flow cytometry

PBMCs were isolated by differential centrifugation over Ficoll-Hypaque, then suspended at 2 × 10^6^/ml in RPMI-1640 and added to two tubes (100 μl in each tube), followed by addition of 5 μl monoclonal antibodies conjugated with different fluorescent dyes, including Vioblue-CD3, PERCP-CD4, PE-cy7-CCR7, FITC-CXCR5, APC-CD45RA and PE-PD-1. Then, 5 μl PE-PD-1 or isotype control was added, followed by incubation for 20 min at 4 °C in dark. The cells were washed with PBS and then centrifugated at 1000 rpm for 5 min. After removing the supernatant, the cells were resuspended in 500 μl PBS. After gating on SSC and FSC, cells with positive expression of CD4 and CD3 were further gated. The CD45RA^−^CXCR5^+^ cells were then gated and CCR7^lo^PD-1^hi^ subset in CD45RA^−^ CXCR5^+^ cells was analyzed. More than 30,000 cells were collected for each sample. Samples were detected by BD flow cytometry (LSR II, BD, USA), and analyzed using FLOWJo software.

### Cytometric bead array

Expression levels of IL-21 and IL-4 were detected by cytometric bead array according to instructions. Briefly, serum sample (50 μl) was mixed with 50 μl of capture microbeads and 50 μl of PE detection reagents (anti-human IL-21 and anti-human IL-4). After incubation for 3 h in the dark at room temperature, samples were washed, rinsed with 300 μl serum enhanced buffer, and then detected. Levels of IL-21 and IL-4 were detected by flow cytometry (LSR II) and the data were analyzed using FCAP software.

### Enzyme-linked immunosorbent assay

Enzyme-linked immunosorbent assay (ELISA) was performed as previously described [8]. Briefly, microtiter plates were coated with 6 μg/ml recombinant antigen B1 of Eg (AgB1) in bicarbonate buffer (pH 9.6) followed by blocking with 5 % nonfat milk in neutral phosphate-buffer saline containing 0.3 % Tween-20 (PBST). Serum samples diluted with PBST were added to antigen-coated wells in duplicate and incubated for 30 min at 37 °C. Positive and negative control sera were also included. After washing with PBST, plates were incubated with monoclonal mouse-anti-human-IgG1 (1:500), -IgG2 (1:50), -IgG3 (1:50), -IgG4 (1:500). HRP-labeled goat-anti-mouse-IgG (1:1000 in PBS) were used as the secondary antibody. Enzyme activity was assayed by incubation for 20 min at room temperature. The positive sera from CE patients served as positive control. The sera from healthy individuals served as negative control. The absorbance values at 450 nm wavelength were detected by a microplate reader. The ODs values of IgGs were normalized to those of negative control and the relative levels of IgGs were compared among different groups.

### In vitro stimulation of PBMCs

PBMCs were isolated from healthy individuals and CE patients with Ficoll. The cell concentration was adjusted to 1 × 10^6^/ml in RPMI-1640. Then, 100 μl PBMCs suspension were added to a 96 well culture plate (1 × 10^5^/well) and cultured for 72 h. Then, PBMCs were co-cultured with 10 ng/ml of IL-6 and 2 μg/ml of soluble anit-CD28 in the presence of HF or PBS at 37 °C with 5 % CO_2_. After incubation for 72 h, cells and the culture supernatants were collected. Percentage of the circulating Tfh cells was detected with flow cytometry. Levels of IL-21 and IL-4 in the culture supernatants were measured by cytometric bead array.

### Statistical analysis

All data were analyzed using SPSS 13.0 software. Results were given as mean ± SD. One-way ANOVA was performed to compare the differences between groups. Correlation analysis was performed with Pearson correlation analysis. *P* value less than 0.05 was considered as statistically significant.

## Results

### The frequency of CCR7^lo^PD-1^hi^ cells within CXCR5^+^ CD4^+^ T cells is increased in CE1, CE2, and CE3 groups

**Fig. 1:**
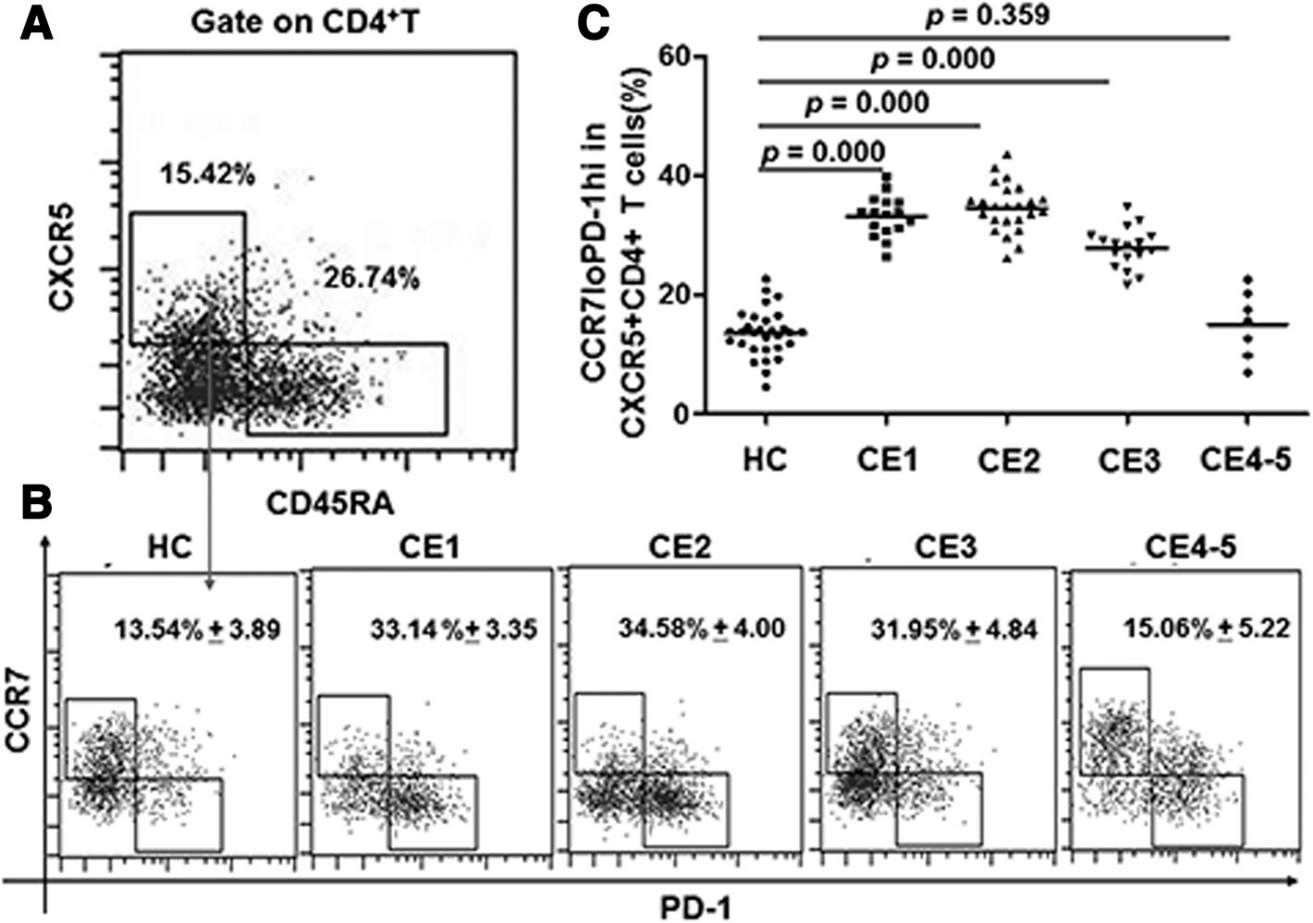
Increased frequency of CCR7^lo^PD-1^hi^ cells within CXCR5^+^ CD4^+^ T cells in CE patients. The PBMCs were stained with Vioblue-CD3, PERCP-CD4, PE-cy7-CCR7, FITC-CXCR5 and APC-CD45RA. The percentages of CCR7^lo^PD-1^hi^ cells within CXCR5^+^CD4^+^ T cells were determined by flow cytometry analysis. **a** Representative flow cytometry dot-plots of the percent of CD45RA^−^CXCR5^+^CD4^+^ T cells. **b** Quantification of phenotypic marker CCR7 and PD-1 in each group. **c** The statistical results of percentage of CCR7^lo^PD-1^hi^ within CXCR5^+^ CD4^+^ T cells in each group

To determine expression of CCR7^lo^PD-1^hi^ T cells in PBMCs from CE patients, flow cytometry analysis was performed. CD45RA was used to identify the effector/memory T cells (CD45RA^−^) in CD3^+^CD4^+^ T cells and the cells positive for CXCR5 was further analyzed for the percentage of Tfh cells expressing CCR7^lo^PD-1^hi^ (Fig. 1). The results showed that the percentages of CCR7^lo^PD-1^hi^ cells within CXCR5^+^ CD4^+^ T cells in CE1 (33.14 % ± 3.35), CE2 (34.58 % ± 4.00), and CE3 (31.95 % ± 4.84) group were significantly increased (*p* < 0.05) compared with those in the healthy controls (13.54 % ± 3.89) and in CE4-5 types (15.06 % ± 5.22). However, difference between CE4-5 group and the healthy control group was not statistically significant. Differences among CE1, CE2, and CE3 groups were also not statistically significant. These results indicated that percentages of CCR7^lo^PD-1^hi^ cells within CXCR5^+^ CD4^+^ T cells are significantly higher in CE1, CE2, and CE3 group patients than in healthy controls.

### Levels of IL-21 and IL-4 in the sera from patients with active disease are elevated

**Fig. 2:**
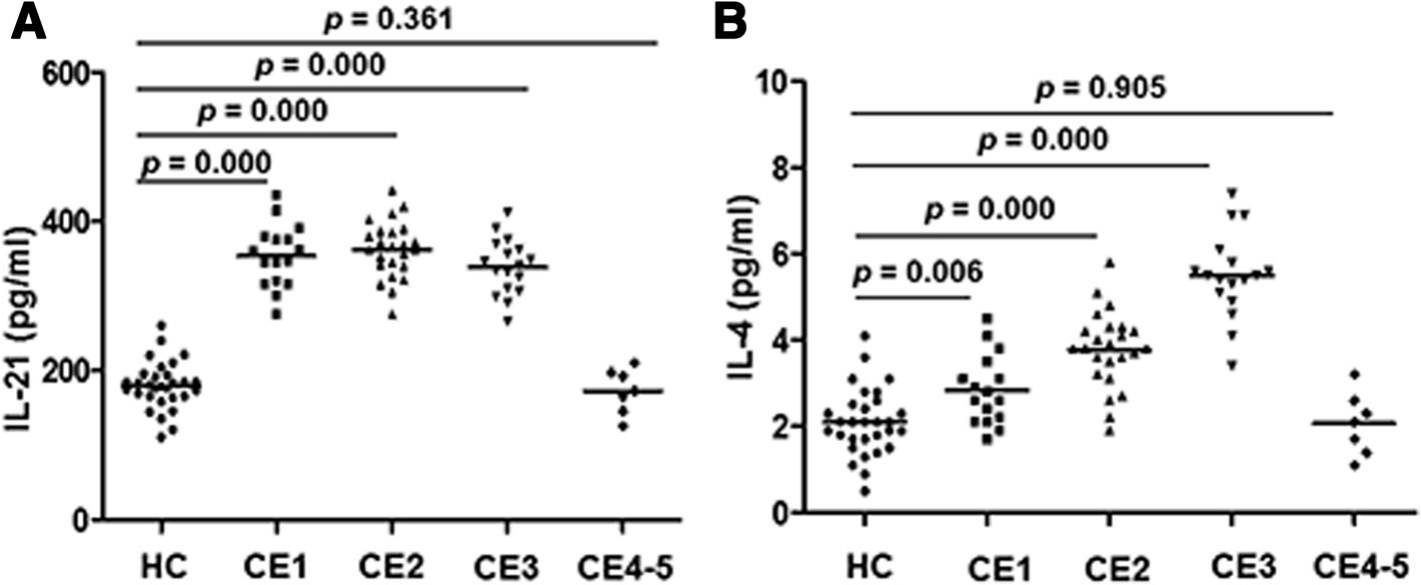
Increased levels of IL-21 and IL-4 in CE patients. Serum samples were incubated with microbeads and detection reagents. The levels of IL-21 and IL-4 in CE were measured by flow cytometry analysis. The differences of IL-21 (**a**) and IL-4 (**b**) in CE were compared

Cytometric bead array was performed to detect levels of IL-21 in serum samples from the patients and healthy controls. The results in Fig. 2 showed that, compared with those in the healthy control group (179.43 ± 31.54 pg/ml), levels of IL-21 were significantly increased in CE1 (351.44 ± 43.78 pg/ml), CE2 (362.17 ± 37.34 pg/ml), and CE3 (338.94 ± 36.73 pg/ml) groups (*p* < 0.05). However, the IL-21 level in CE4-5 (193.57 ± 21.08 pg/ml) was not different from that in healthy controls. The difference among CE1, CE2, and CE3 types was not significant.

We also measured the levels of IL-4 in sera from patients in comparison with healthy controls. As shown in Fig. 2b, levels of IL-4 in CE1 (2.84 ± 0.78 pg/ml), CE2 (3.78 ± 0.87 pg/ml), and CE3 (5.5 ± 0.96 pg/ml) groups were significantly increased compared with those in the healthy control group (2.1 ± 0.76 pg/ml) and CE4-5 (2.06 ± 0.67 pg/ml) group (CE1, *p* < 0.05; CE2, *p* < 0.05; CE3, *p* < 0.05). The difference between CE4-5 and the healthy controls was not statistically significant. The level of IL-4 in CE2 was significantly increased compared with that in CE1 (*p* < 0.05). Level of IL-4 in CE3 group was significantly increased compared with those in CE2 and CE1 (*p* < 0.05, *p* < 0.05). Together, the above results indicated that levels of both IL-21 and IL-4 are increased in CE1, CE2, CE3 patients when compared with those in the healthy controls and CE4-5 patients.

### Changes in the transcripts of IL-21, IL-4, Bcl-6, and Blimp-1 in CE patients

**Fig. 3:**
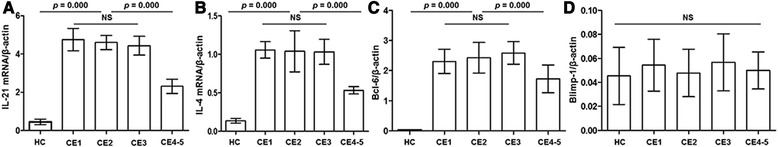
Differential mRNA expression of IL-21, IL-4, Bcl-6, and Blimp-1 in PBMCs of CE patients. The expression of the mRNA levels of cytokines and transcription factors were measured in total RNA isolated from the PBMCs by real-time RT-PCR. β-actin was used as an internal control. The mean and SD were shown for each group. **a** The expression of IL-21 mRNA. **b** The expression of IL-4 mRNA. **c** The expression of Bcl-6 mRNA. **d** The expression of Blimp-1 mRNA

The mRNA levels of IL-21, IL-4, Bcl-6, and Blimp-1 were determined by real-time PCR. As shown in Fig. 3, results showed that the expression levels of IL-21, IL-4, and Bcl-6 were significantly increased in CE1, CE2, and CE3 compared with those in the healthy control group and CE4-5 (*p* < 0.05). The differences among CE1, CE2, and CE3 groups were not statistically significant. The mRNA levels of Blimp-1 in CE types were relatively lower in comparison with those of IL-21, IL-4, Bcl-6. And the difference was not statistically significant. These results indicated that the mRNA levels of IL-21, IL-4, and Bcl-6 are increased in progress of CE.

### Changes in relative levels of antibodies in the progress of CE

Since our results so far demonstrated that the percentages of Tfh cells and the levels of cytokines and transcription factors associated with Tfh cells in the peripheral blood of CE patients (CE1-3) were increased compared with those in CE4-5 and healthy controls, and Tfh cells are known to influence the immunoglobulin class switching, we studied the changes in relative antibody levels and IgG subclasses in CE patients at different disease status with ELISA.

**Fig. 4:**
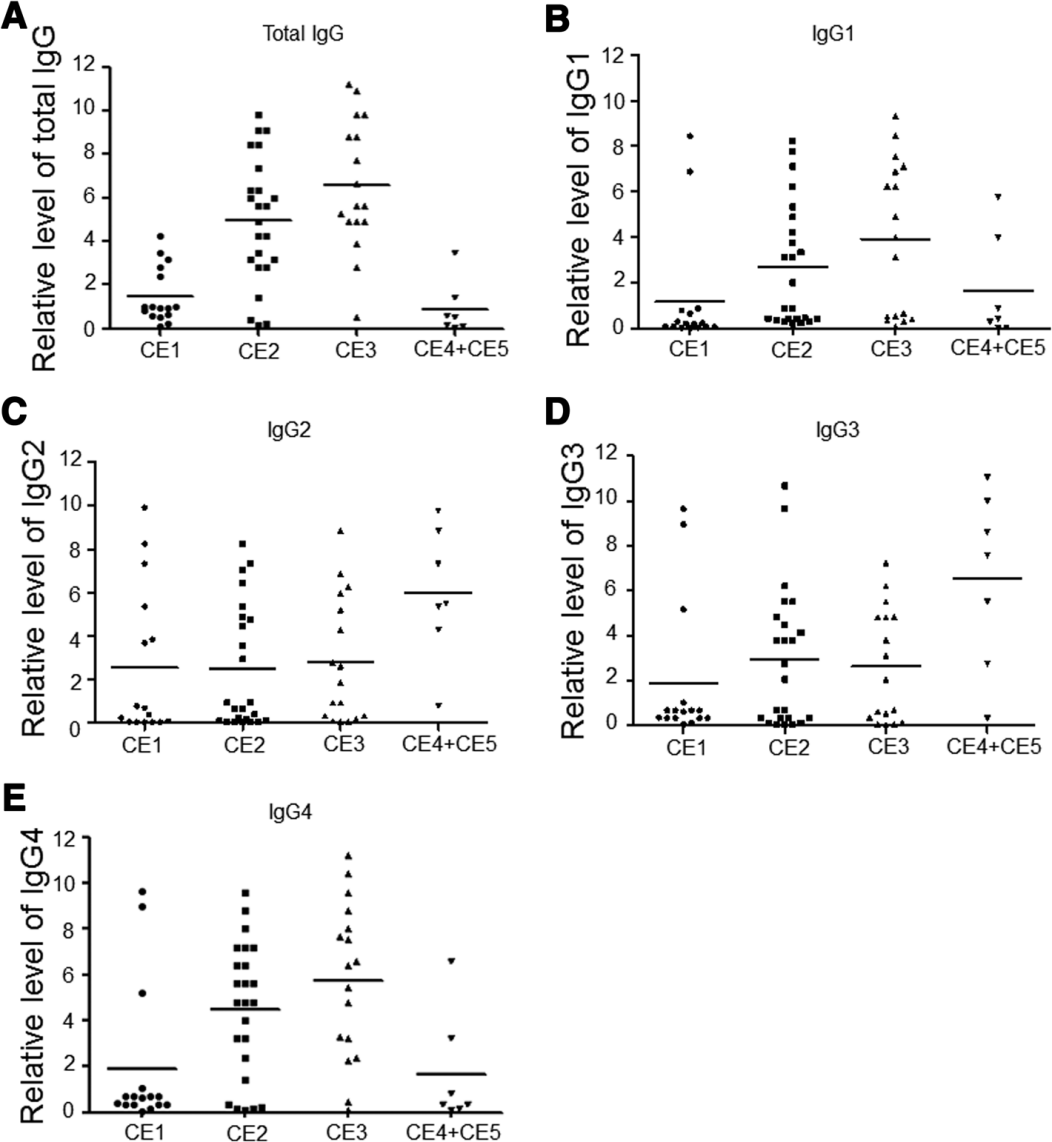
The relative levels of IgG and subclasses in CE patients. E.granulosus antigen B-specific total IgG and IgG subclasses in the serum samples were measured by ELISA. Means and SD were shown for each group. Different levels of IgG, IgG1, IgG2, IgG3, and IgG4 in CE patients were compared. OD values of these IgGs in CE patients were normalized to those in healthy controls (negative control). **a** The relative level of total IgG in CE patients’ sera. **b** The relative level of IgG1 in CE patients’ sera. **c** The relative level of IgG2 in CE patients’ sera. **d** The relative level of IgG3 in CE patients’ sera. **e** The relative level of IgG4 in CE patients’ sera

The relative levels of total IgG and subclasses (Fig. 4) were changed in CE patients. The relative levels of total IgG were increased in the CE1, CE2, and CE3 patients, with CE3 patients have the highest relative level. Total IgG relative levels in patients with CE1, CE2, CE3 were higher than that in patients with CE4-5 and healthy controls.

In the Eg infected patients, IgG1 and IgG4 were mainly increased in patients with CE1, CE2, and CE3. IgG1 and IgG3 in the patients with CE1, CD2 and CE3 were higher than that of healthy controls, and the peak of IgG1 and IgG4 appeared at CE3. IgG1 and IgG4 relative levels in CE1, CE2 and CE3 patients were higher than the healthy controls and CE4-5 patients.

The relative levels of IgG2 and IgG3 in CE4-5 patients were higher than that in CE1, CE2 and CE3 patients, and the IgG2 and IgG3 in Eg infected patients (CE1-CE5) were increased compared with healthy controls, which was in consistent with increased Tfh cytokines and transcription factors observed in PBMC of patients.

### Levels of IL-21 in CE1, CE2, and CE3 are positively correlated with number of CCR7^lo^PD-1^hi^ cells within CXCR5^+^ CD4^+^ T cells in CE patients

To determine correlation between levels of IL-21 and IL-4 and the number of CCR7^lo^PD-1^hi^ cells within CXCR5^+^ CD4^+^ T cells (circulating Tfh cells) in patients with CE, we performed Pearson correlation analysis. The results in Table 3 showed that the levels of IL-21 in CE1 (r = 0.529), CE2 (r = 0.551), and CE3 (r = 0.779) was positively correlated with percentages of circulating Tfh cells. However, the levels of IL-4 in all groups were not correlated with circulating Tfh cells. These results suggest that levels of IL-21, but not IL-4, in CE1, CE2, and CE3 patients are positively correlated with percentages of circulating Tfh cells.

**Table 3:** Correlations between circulating Tfh, IL-4, and IL-21

Circulating Tfh			IL-4	IL-21
	CE1	r	−0.004	0.529
		P	>0.05	< 0.05
	CE2	r	−0.06	0.551
		P	> 0.05	< 0.05
	CE3	r	−0.169	0.779
		P	> 0.05	< 0.05
	CE4-5	r	−0.536	0.071
		P	> 0.05	> 0.05

### Levels of circulating Tfh cells, IL-21 and IL-4 are increased after in vitro stimulation with HF

**Fig. 5:**
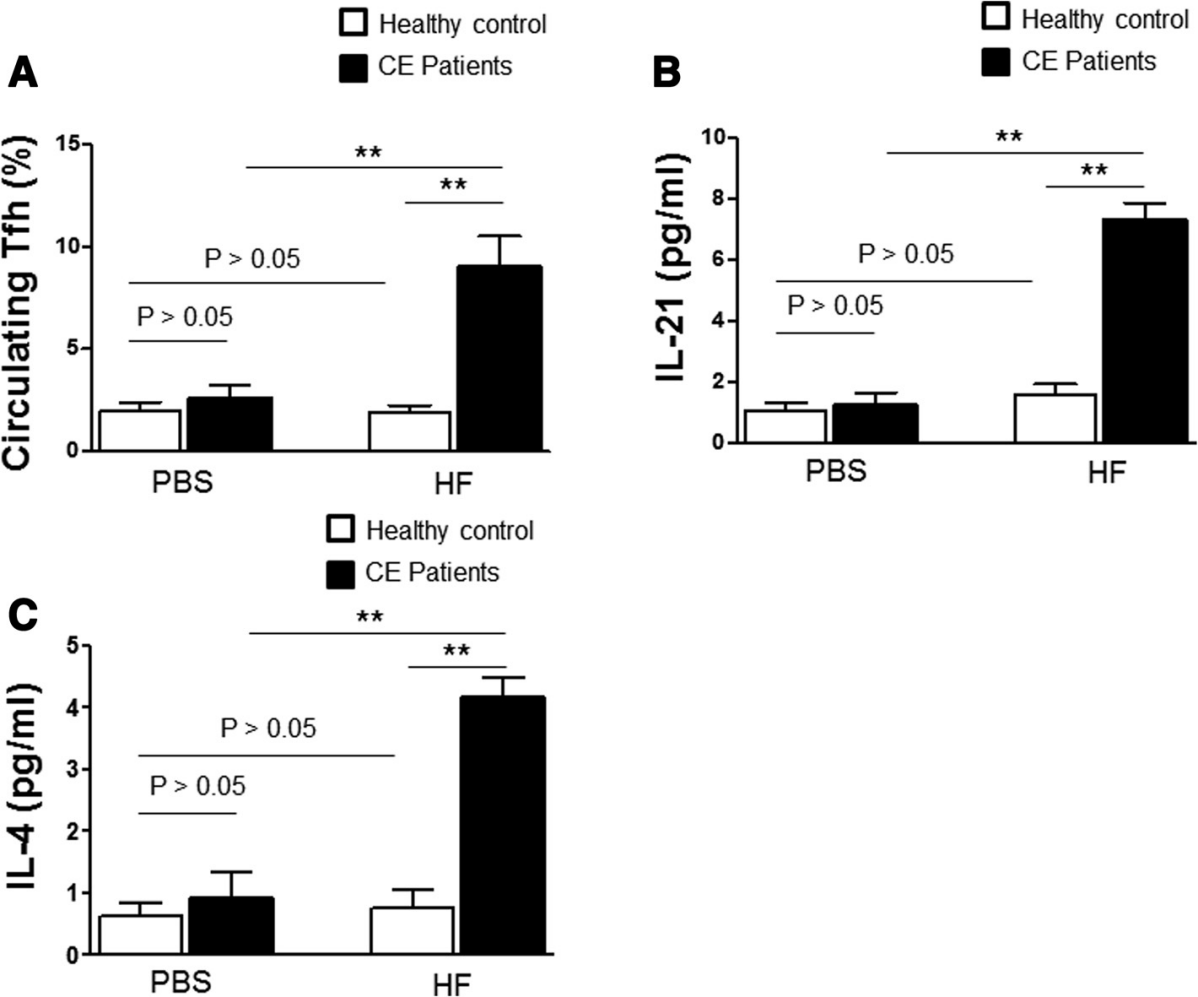
Analysis of circulating Tfh cells and IL-21 levels after in vitro stimulation. PBMCs were isolated from healthy individuals and CE patients. Then they were stimulated with PBS or HF in vitro. The percentage of circulating Tfh cells was detected with flow cytometry. The levels of IL-21 and IL-4 were measured with cytometric bead array. **a** The percentage of circulating Tfh cells. **b** Level of IL-21 in the culture supernatant. **c** Level of IL-4 in the culture supernatant

To detect the levels of circulating Tfh cells, IL-21 and IL-4 after in vitro stimulation with HF, we co-cultured PBMCs isolated from healthy controls and CE patients with HF or PBS. As shown in Fig. 5a, the percentage of circulating Tfh cells was not increased after in vitro stimulation with PBS. However, after stimulation with HF, the percentage of circulating Tfh cells was significantly higher in the PBMCs isolated from CE patients than that from healthy individuals (*p* < 0.05). As shown in Fig. 5b and Fig. 5c, PBS treatment did not increase the levels of IL21 and IL-4 in culture supernatant. In contrast, HF treatment increased the levels of IL21 and IL-4 in culture supernatant. The levels of IL-21 and IL-4 in the supernatant were significantly higher in the PBMCs from CE patients than those from healthy individuals (*p* < 0.05). Thus, after stimulating PBMCs from CE patients with HF in vitro, levels of circulating Tfh cells, IL-21 and IL-4 are increased.

## Discussion

Similar to infections with other parasites in humans, *Eg* evolves to obtain immune evasion capacity during the chronic interaction with its host immunity. Studies with animal models and clinical observations of humans infected with hydatid diseases suggest that the host immunity is dominated by Th2 cells, which mainly produces IL-4 with the increase of parasitic burden at the end stage of the disease and is detrimental to the host protective immunity against parasite infection [1, 13]. Moreover, antibody class switching is obviously triggered at the advanced stage of hydatid infection. The subclass of IgG is different in different types of CE [7]. It is reported that the Tfh cells influence the type and affinity of antibody production during infection [29–31]. Our current study demonstrated that Tfh cell numbers increased in patients with CE1-3 but decreased in CE4-5 patients. In correlation with this, the major Tfh cytokine IL-21 and IL-4 and transcription factors Bcl-6 was also increased at the mRNA levels in the PBMCs of patients with CE1-3 but not CE4-5. The IgG subtype, levels of IgG1 and IgG4 were increased in patients with CE1-3 and that of IgG2 and IgG3 was increased in patients with CE4-5. Together these data suggest that Tfh cells in the peripheral blood of hydatid infection change with diseases severity and are correlated with changes in IgG subtype specific to certain diseases spectra.

Compared with healthy controls, the frequency of peripheral blood circulating Tfh cells was increased in CE1, CE2, and CE3 patients. It is reported that circulating Tfh cells is significantly increased in peripheral blood of systemic lupus erythematosus, rheumatoid arthritis, and human immunodeficiency virus patients, and was rapidly increased in the vaccinated people [19, 20, 22]. In other parasite infection, Tfh cells are also increased [23, 24]. The in vitro co-culture of PBMCs from CE patients with HF induced the differentiation of circulating Tfh cells in the present study. All these results showed that circulating Tfh cells were significantly increased in peripheral blood of CE, indicating that circulating Tfh cells are involved in the immune response to CE infection.

We also demonstrated that concentrations of IL-21 and IL-4 in peripheral blood were increased in CE1, CE2, CE3, and those of patients with CE4-5 returned to the levels close to healthy controls. IL-21 is critical for the function of Tfh cells [18, 21], and it helps B cells to produce high titer and high affinity antibodies against their cognate antigens. B cells enter into follicle to form germinal center and finally differentiate into the long-acting plasma cells in T cell-dependent immune responses [31]. Moreover, IL-21 produced by Tfh cells promotes the long-lasting plasma cells to produce high affinity antibodies [32–34]. IL-21 cells play an important role in the formation of Tfh cells and in the differentiation, growth, survival, and transformation of B cells as well [35, 36]. IL-4 is another cytokine produced by Tfh cells and it is mainly produced by Tfh cells in the lymph nodes of patients infected with parasite [37]. During the chronic parasitic infection, IL-4 is indispensable for proliferation of Th2 and B cells. B cells promote Tfh cells in the germinal center to produce IL-4 and IL-21. These two complementary cytokines are critical for differentiation of Tfh cells while have little effect on the germinal center formation [38, 39]. During the process of infection, expression level of IL-21 produced by Tfh cells is much higher than IL-4 [40].

In the present study, levels of IL-21 and IL-4 were increased in the active stage of CE, and level of IL-21 was positively correlated with percentage of circulating Tfh cells in the patients with active and transitional CE. However, IL-4 level did not correlate with percentage of circulating Tfh cells. Taken together, these results indicate that IL-21 and IL-4 are involved in the immune responses against hydatid infection. Furthermore, the mRNA levels of IL-21, IL-4, and Bcl-6 were increased in CE1, CE2 and CE3. As the transcription factor of germinal center Tfh cells, Bcl-6 is up-regulated in Tfh cells and plays an important role in proliferation of GC Tfh cells [41]. Blimp-1 inhibits expression of Bcl-6 in T cells and promotes T cells to differentiate into other T cell subtypes. Only Bcl-6 could determine the differentiation of germinal center Tfh cells [27, 42, 43]. It is reported that the mRNA level of Bcl-6 is moderately expressed in systemic lupus erythematosus and rheumatoid arthritis, but the mRNA level of Blimp-1 is very low [29, 30]. In the present study, the mRNA level of Bcl-6 was increased in patients with active and transitional CE infection, but the mRNA level of Blimp-1 was very low in the same patients. It is demonstrated that Tfh cells originated in the T-B border region and follicles of lymph nodes may circulate into the peripheral blood and form the Tfh cell compartment [28]. The activated T cells with characteristics of Tfh cells are closely related with the immune responses [18, 25, 26, 44]. Different from cells in lymph nodes, Tfh cells in peripheral blood are in a quiescent or memory state [25, 44]. Bcl-6 promotes proliferation of T cells [44–46]. Bcl-6 is expressed in Tfh cells at early differentiation in the T-B border, as well as in Tfh cells in the follicles of lymph nodes [47–51].

In the progress of CE disease, two types of antigens are exposed to the host immune system and induce the production of antibodies. Laminated layer materials may contribute to the T-independent antibody generation by active B cells. Our results indicate that *Eg* infection up-regulate the mRNA level of Bcl-6 in T cells (Additional file 1: Figure S1), and the Bcl-6 protein may not be expressed in these peripheral blood Tfh cells because that these Tfh cells in the peripheral blood coming from the secondary lymphoid organs may express the mRNA but not protein of Bcl-6 [52–55]. Hence we did not study the expression of Bcl-6 protein in peripheral blood Tfh cells in the current study.

Our results also demonstrated that level of total IgG was increased in CE1, CE2, and CE3, and levels of IgG1, IgG4 was increased in CE1, CE2, and CE3. This is consistent with the previous reports. They reported that level of IgG4 was increased in the active and transitional CE, and that the seroprevalence of IgG4 was increased whereas the levels of IgG1 and IgG4 were decreased in CE4–5 [7, 9, 56].

## Conclusion

Our study found that the circulating Tfh cells and related cytokines were closely related to the cyst stage. Circulating Tfh cells not only involve in the immune responses initiated by *Eg*-infection, but also may be associated with IgG class switching during hydatid infection.

## Additional file

Additional file 1: Figure S1. The schematic of circulating Tfh cells and antibody formation in peripheral blood of CE. *Eg* can release a large amount of Ag which may active Naive T cells by APC (antigen-present-cell). Naive T cells express Bcl-6 and differentiate into effector T cells. Most Bcl-6 high expression effector T cells develop into Tfh cells to help B cells to produce antibodies in host *Eg*-infection. Some of Bcl-6 high expression effector T cells move into peripheral blood and develop into circulating Tfh cells, which can produce IL-21. (TIFF 188 kb)

## Abbreviations

Tfh: T follicular helper cystic echinococcosis
CE: Cystic echinococcosis
*Eg*: *Echinococcus granulosus*
PBMC: Peripheral blood mononuclear cells
ELISA: Enzyme-linked immunosorbent assay
AgB: Antigen B of Eg
PBST: Phosphate-buffer saline containing 0.3 % Tween-20
