# Supplementary material for: CCR7loPD-1hi CXCR5+ CD4+ T cells are positively correlated with levels of IL-21 in active and transitional cystic echinococcosis patients
Source: BMC Infect Dis. 2015 Oct 26;15:457. doi: 10.1186/s12879-015-1156-9 (PMC4624577; doi:10.1186/s12879-015-1156-9)
Supplement: Additional file 1: Figure S1. — The schematic of circulating Tfh cells and antibody formation in peripheral blood of CE. Eg can release a large amount of Ag which may active Naive T cells by APC (antigen-present-cell). Naive T cells express Bcl-6 and differentiate into effector T cells. Most Bcl-6 high expression effector T cells develop into Tfh cells to help B cells to produce antibodies in host Eg-infection. Some of Bcl-6 high expression effector T cells move into peripheral blood and develop into circulating Tfh cells, which can produce IL-21. (TIFF 188 kb) [file 12879_2015_1156_MOESM1_ESM.tiff]

**Table 1** Information of the patients enrolled in this study.

| Case | Stages | Age (range) | Gender | Position | Size (cm) | Populations |
| --- | --- | --- | --- | --- | --- | --- |
| 1 | CE1 | 21-25 | male | left liver lobe | 4.5 | han |
| 2 | CE1 | 21-25 | male | right liver lobe | 4.1 | han |
| 3 | CE1 | 21-25 | male | left liver lobe | 4.3 | han |
| 4 | CE1 | 31-35 | female | right liver lobe | 5.6 | Uighur |
| 5 | CE1 | 36-40 | female | right liver lobe | 4.9 | kazak |
| 6 | CE1 | 26-30 | male | right liver lobe | 5.6 | Uighur |
| 7 | CE1 | 26-30 | male | right liver lobe | 5.8 | Uighur |
| 8 | CE1 | 21-25 | female | left liver lobe | 4.6 | han |
| 9 | CE1 | 21-25 | male | left liver lobe | 3.9 | kazak |
| 10 | CE1 | 36-40 | male | left liver lobe | 6.8 | Uighur |
| 11 | CE1 | 26-30 | female | right liver lobe | 4.9 | Uighur |
| 12 | CE1 | 31-35 | male | left liver lobe | 5.9 | Uighur |
| 13 | CE1 | 31-35 | female | right liver lobe | 6.9 | han |
| 14 | CE1 | 36-40 | male | right liver lobe | 7.9 | Uighur |
| 15 | CE1 | 41-45 | male | right liver lobe | 6.8 | Uighur |
| 16 | CE1 | 36-40 | male | right liver lobe | 5.9 | kazak |
| 17 | CE2 | 36-40 | female | right liver lobe | 7.9 | kazak |
| 18 | CE2 | 36-40 | male | left liver lobe | 4.9 | han |
| 19 | CE2 | 46-50 | male | left liver lobe | 4.8 | kazak |
| 20 | CE2 | 31-35 | male | right liver lobe | 6.9 | kazak |
| 21 | CE2 | 36-40 | male | left liver lobe | 4.8 | Uighur |
| 22 | CE2 | 31-35 | male | right liver lobe | 7.9 | han |
| 23 | CE2 | 21-25 | female | right liver lobe | 8.7 | han |
| 24 | CE2 | 31-35 | male | left liver lobe | 8.9 | han |
| 25 | CE2 | 26-30 | male | right liver lobe，left liver lobe | 8.6 | kazak |
| 26 | CE2 | 26-30 | male | left liver lobe | 8.9 | han |
| 27 | CE2 | 31-35 | male | right liver lobe | 8.7 | kazak |
| 28 | CE2 | 26-30 | female | right liver lobe | 9.4 | han |
| 29 | CE2 | 36-40 | female | left liver lobe | 10.6 | han |
| 30 | CE2 | 31-35 | male | right liver lobe | 11.9 | han |
| 31 | CE2 | 26-30 | female | left liver lobe | 15.6 | khalkhas |
| 32 | CE2 | 26-30 | male | right liver lobe | 16.3 | kazak |
| 33 | CE2 | 31-35 | male | left liver lobe | 18.9 | khalkhas |
| 34 | CE2 | 36-40 | female | right liver lobe | 19.6 | kazak |
| 35 | CE2 | 26-30 | male | right liver lobe | 20.9 | han |
| 36 | CE2 | 26-30 | female | right liver lobe | 21.6 | khalkhas |
| 37 | CE2 | 21-25 | female | right liver lobe | 12.3 | kazak |
| 38 | CE2 | 26-30 | male | left liver lobe | 14.4 | Uighur |
| 39 | CE2 | 31-35 | male | left liver lobe | 7.6 | Uighur |
| 40 | CE2 | 31-35 | female | right liver lobe | 5.6 | Uighur |
| 41 | CE3 | 45-50 | female | left liver lobe | 7.8 | khalkhas |
| 42 | CE3 | 41-45 | female | right liver lobe | 19.3 | khalkhas |
| 43 | CE3 | 36-40 | male | left liver lobe | 12.3 | Uighur |
| 44 | CE3 | 36-40 | female | right liver lobe | 14.2 | Uighur |
| 45 | CE3 | 31-35 | male | right liver lobe, left liver lobe | 8.4 | han |
| 46 | CE3 | 26-30 | male | left liver lobe | 9.2 | tibetan |
| 47 | CE3 | 36-40 | male | left liver lobe | 8.6 | han |
| 48 | CE3 | 31-35 | female | left liver lobe | 7.6 | kazak |
| 49 | CE3 | 26-30 | male | right liver lobe | 6.4 | han |
| 50 | CE3 | 26-30 | male | right liver lobe | 12.3 | Uighur |
| 51 | CE3 | 36-40 | male | right liver lobe | 11.4 | Uighur |
| 52 | CE3 | 31-35 | male | right liver lobe | 8.3 | han |
| 53 | CE3 | 31-35 | male | right liver lobe | 6.8 | Uighur |
| 54 | CE3 | 31-35 | male | right liver lobe | 11.6 | kazak |
| 55 | CE3 | 31-35 | female | right liver lobe | 6.8 | han |
| 56 | CE3 | 36-40 | female | right liver lobe | 7.9 | han |
| 57 | CE3 | 36-40 | male | right liver lobe | 8.6 | han |
| 58 | CE4-5 | 36-40 | male | right liver lobe | 6.9 | han |
| 59 | CE4-5 | 41-45 | male | right liver lobe | 8.7 | han |
| 60 | CE4-5 | 41-45 | male | right liver lobe | 10.6 | mongolian |
| 61 | CE4-5 | 41-45 | male | right liver lobe | 5.6 | mongolian |
| 62 | CE4-5 | 36-40 | male | right liver lobe | 4.6 | Uighur |
| 63 | CE4-5 | 36-40 | female | right liver lobe | 4.8 | mongolian |
| 64 | CE4-5 | 41-45 | male | right liver lobe | 9.4 | mongolian |

**Table 2** Primers used in this study.

| Primers | Sequences (5' to 3') |
| --- | --- |
| IL-4_F | TTTGCTGCCTCCAAGAACAC |
| IL-4_R | TTCCTGTCGAGCCGTTTCAG |
| IL-21_F | ACACAGACTAACATGCCCTTCA |
| IL-21_R | ACCGTGAGTAACTAAGAAGCAAATC |
| BCL-6_F | GGAAACCCAGTCAGAGTATTCG |
| BCL-6_R | CACATTTGTAGGGCTTTTCTCC |
| β-actin_F | TAGGCGGACTGTTACTGAGC |
| β-actin_R | TGCTCCAACCAACTGCTGTC |
| Blimp-1_F | TCCAGCACTGTGAGGTTTCA |
| Blimp-1_R | TCAAACTCAGCCTCTGTCCA |

**Table 3** Correlations between circulating Tfh, IL-4, and IL-21.

| Circulating Tfh |  |  | IL-4 | IL-21 |
| --- | --- | --- | --- | --- |
|  | CE1 | r | -0.004 | 0.529 |
|  |  | P | > 0.05 | < 0.05 |
|  | CE2 | r | -0.06 | 0.551 |
|  |  | P | > 0.05 | < 0.05 |
|  | CE3 | r | -0.169 | 0.779 |
|  |  | P | > 0.05 | < 0.05 |
|  | CE4-5 | r | -0.536 | 0.071 |
|  |  | P | > 0.05 | > 0.05 |
